# Zinc-modified titanium surface enhances osteoblast differentiation of dental pulp stem cells *in vitro*

**DOI:** 10.1038/srep29462

**Published:** 2016-07-08

**Authors:** Kazuyuki Yusa, Osamu Yamamoto, Hiroshi Takano, Masayuki Fukuda, Mitsuyoshi Iino

**Affiliations:** 1 Department of Dentistry, Oral and Maxillofacial-Plastic and Reconstructive Surgery, School of Medicine, Yamagata University, Yamagata, Japan; 2 Department of Bio-System Engineering, Graduate School of Science and Engineering, Yamagata University, Yonezawa, Japan; 3 Division of Dentistry and Oral Surgery, Akita University School of Medicine, Akita, Japan

## Abstract

Zinc is an essential trace element that plays an important role in differentiation of osteoblasts and bone modeling. This *in vitro* study aimed to evaluate the osteoblast differentiation of human dental pulp stem cells (DPSCs) on zinc-modified titanium (Zn-Ti) that releases zinc ions from its surface. Based on real-time PCR, alkaline phosphatase (ALP) activity and Western blot analysis data, we investigated osteoblast differentiation of DPSCs cultured on Zn-Ti and controls. DPSCs cultured on Zn-Ti exhibited significantly up-regulated gene expression levels of osteoblast-related genes of type I collagen (Col I), bone morphogenetic protein 2 (BMP2), ALP, runt-related transcription factor 2 (Runx2), osteopontin (OPN), and vascular endothelial growth factor A (VEGF A), as compared with controls. We also investigated extracellular matrix (ECM) mineralization by Alizarin Red S (ARS) staining and found that Zn-Ti significantly promoted ECM mineralization when compared with controls. These findings suggest that the combination of Zn-Ti and DPSCs provides a novel approach for bone regeneration therapy.

Titanium (Ti) and its alloys are widely used in the fields of dentistry and oral and maxillofacial surgery, such as bone fixation devices and dental implants, mainly due to their excellent mechanical properties, superior biocompatibility, stability, and osseointegration potential^1^,^2^,^3^. They have also been used for bone regeneration therapies such as the TIME technique, which is characterized by the use of micro-titanium augmentation mesh, in maxilla and mandibular bone reconstruction after tumor resection^4^,^5^,^6^. Although this therapy is generally successful, some failures still occur due to surgical site infection or deficient bone formation on the surface of Ti. Hence, there have been numerous attempts at Ti surface modification in order to induce rapid bone growth based on osteogenesis, bone modeling and inhibition of bone resorption^7^,^8^,^9^. Alkali heat treatment is one of the most successful and acceptable treatments to develop a modified Ti surface that induces bone formation^10^. Zinc is an essential trace element that has been confirmed to have direct effects on osteoblast differentiation and bone tissue development. Zinc also acts as a signaling molecule and influences intracellular signaling pathways^11^,^12^ and has been shown to have a prominent effect on osteogenesis, due to the induction alkaline phosphatase synthesis and collagen synthesis^13^,^14^,^15^,^16^,^17^,^18^,^19^. Hence, zinc-deficient conditions affect downregulation of alkaline phosphatase activity, osteoblast marker gene expression and decreasing calcium deposition and bone growth *in vitro* and *in vivo*^20^,^21^. In a previous study, we developed a zinc ion-containing alkali heat treatment to fabricate zinc-modified titanium implants. The implant showed significant effects on osteoblast differentiation and bone formation, as it releases zinc ions from its surface, and shear strength between the bone and zinc-modified titanium surface was 5-fold greater than that of the non-zinc modified titanium implant^22^. In addition, the Ti surface-modified technique is also acceptable for bone regeneration therapies as a growth factor-releasing scaffold.

Bone regeneration therapies also need stem cell sources for differentiation into osteoblasts and osteocytes. Embryonic stem (ES) cells and induced pluripotent stem (iPS) cells are able to differentiate into various cell types *in vitro* and *in vivo*^23^,^24^,^25^,^26^,^27^,^28^, and may be a stem cell source for bone regeneration therapy^29^,^30^,^31^,^32^,^33^,^34^. In addition, bone marrow-derived mesenchymal stem cells (BMSCs) are also a stem cell source for bone regeneration therapies and are widely used in both *in vitro* and *in vivo* investigations, as well as for clinical applications^35^,^36^. However, it has been noted that the population of BMSCs is exceedingly small (about 0.001–0.01%)^37^, and their extraction requires invasive and painful procedures for bone marrow aspiration. Dental pulp stem cells (DPSCs) are somatic stem cells from dental pulp tissue that also contain fibroblasts, collagen fibers, nerves, blood vessels with histiocytes, macrophages, mast cells, and plasma cells. The characteristics of DPSCs are similar to those of BMSCs; both are able to differentiate into osteoblasts, chondrocytes, and adipocytes under differentiation-inducing conditions^38^. Furthermore, other reports have noted that DPSCs were found to be multipotent with a high osteoblast potential when compared with BMSCs^39^.

The present *in vitro* study utilizes zinc-modified Ti (Zn-Ti) as a growth factor-releasing scaffold and DPSCs as a stem cell source for bone regeneration therapy. Evaluation of osteoblast differentiation and matrix mineralization was performed, and the results confirmed prominent effects of Zn-Ti on osteoblast differentiation of DPSCs.

## Results

### Surface roughness and contact angle of Zn-Ti

**Figure 1:**
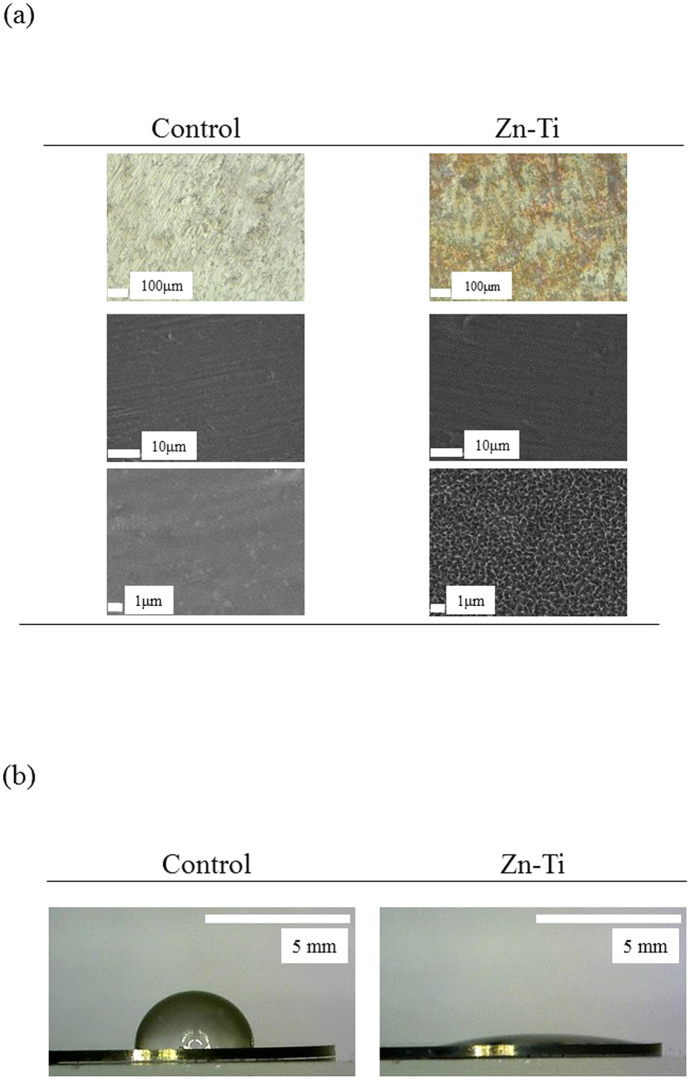
Surface topography and contact angle images of Zn-Ti and control. (**a**) Confocal laser scanning microscope (upper panel) and FE-SEM (middle and lower panel) photographs of Zn-Ti plate surface showing nanoscale porous structure. (**b**) Contact angles of Zn-Ti were lower than those of controls.

Surface characteristics of Zn-Ti were as reported previously^21^. FE-SEM and confocal laser scanning microscope photographs of the surfaces of the Zn-Ti showed a nanoscale porous structure (Fig. 1a). Surface roughness of control and Zn-Ti plates was 0.20 ± 0.04 μm and 0.33 ± 0.05 μm, respectively (Table 1). To evaluate the properties of titanium surface, wettability measurement is a typical strategy for assessing the hydrophilicity of material surfaces. When compared with controls, Zn-Ti was more hydrophilic owing to the surface treatment using an alkali solution containing the [Zn(OH)_4_]^2−^ complex (Fig. 1b, Table 1).

### Zn ion release

**Figure 2:**
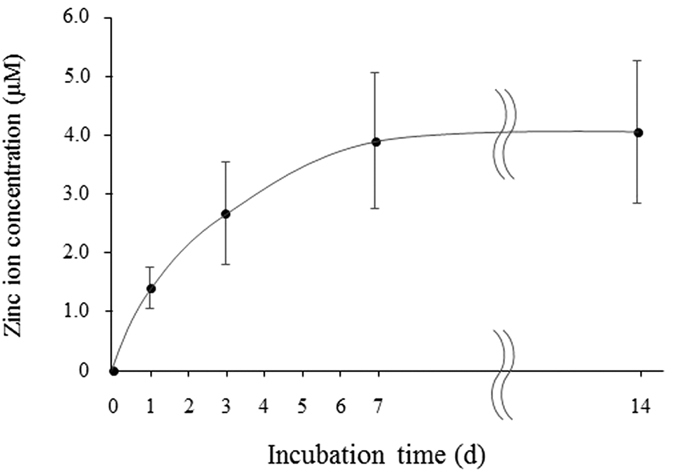
Zinc ion concentrations measured by ICP-AES.

One of the most important characteristics when considering the zinc ion-releasing surface treatment is the release rate. Figure 2 shows the total amount of zinc ions released from the Zn-Ti into PBS. As shown in Fig. 2, zinc ion concentrations were 1.43 ± 0.36 μM, 2.73 ± 0.89 μM, 3.99 ± 1.18 μM and 4.14 ± 1.25 μM on days 1, 3, 7, and 14 respectively.

### Cell culture and FACS

**Figure 3:**
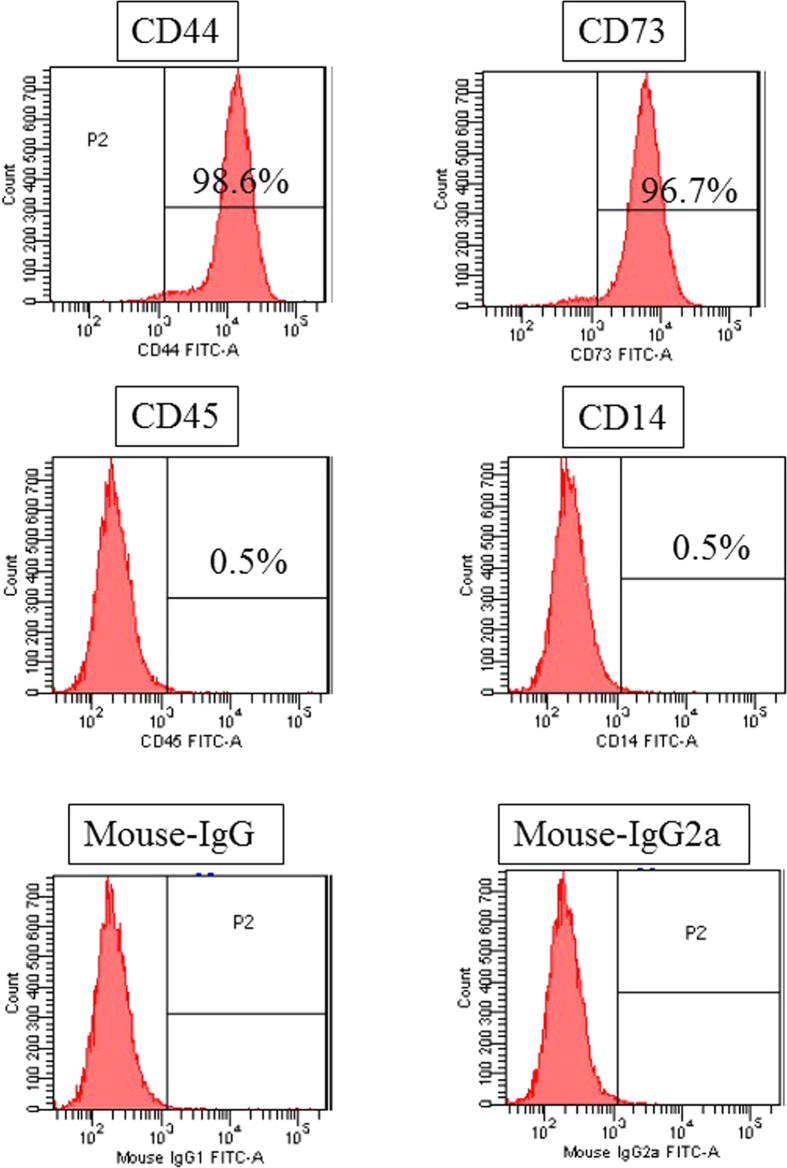
Characterization and flow cytometric analysis of DPSCs. DPSCs were positive for MSC markers CD44 and CD73 and were negative for hematopoietic markers CD45 and CD14.

In order to characterize DPSCs, immunophenotype profiling for specific cell surface antigen sets for MSCs was performed at passage 3 using flow cytometry. In DPSCs, mesenchymal markers such as CD44 and CD73 were positive, and hematopoietic markers such as CD45 and CD14 were negative (Fig. 3). This pattern of expression is commonly found in MSCs.

### Cell proliferation assay

**Figure 4:**
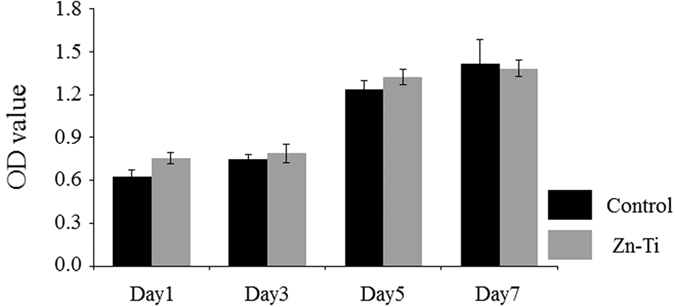
Results of cell proliferation assays. Data represents means ± SD (n = 4, four replicates per time point for each experimental condition). There were no significant differences between Zn-Ti and control plates at each time point.

Cell number was determined on the control and Zn-Ti plates up to 7 days of culture (Fig. 4). Culture growth reached a plateau at day 5. From days 1 to 5, the number of cells was higher on Zn-Ti than on controls, but the differences were not significant.

### Real-time PCR analysis of osteoblast- and odontoblast related genes

**Figure 5:**
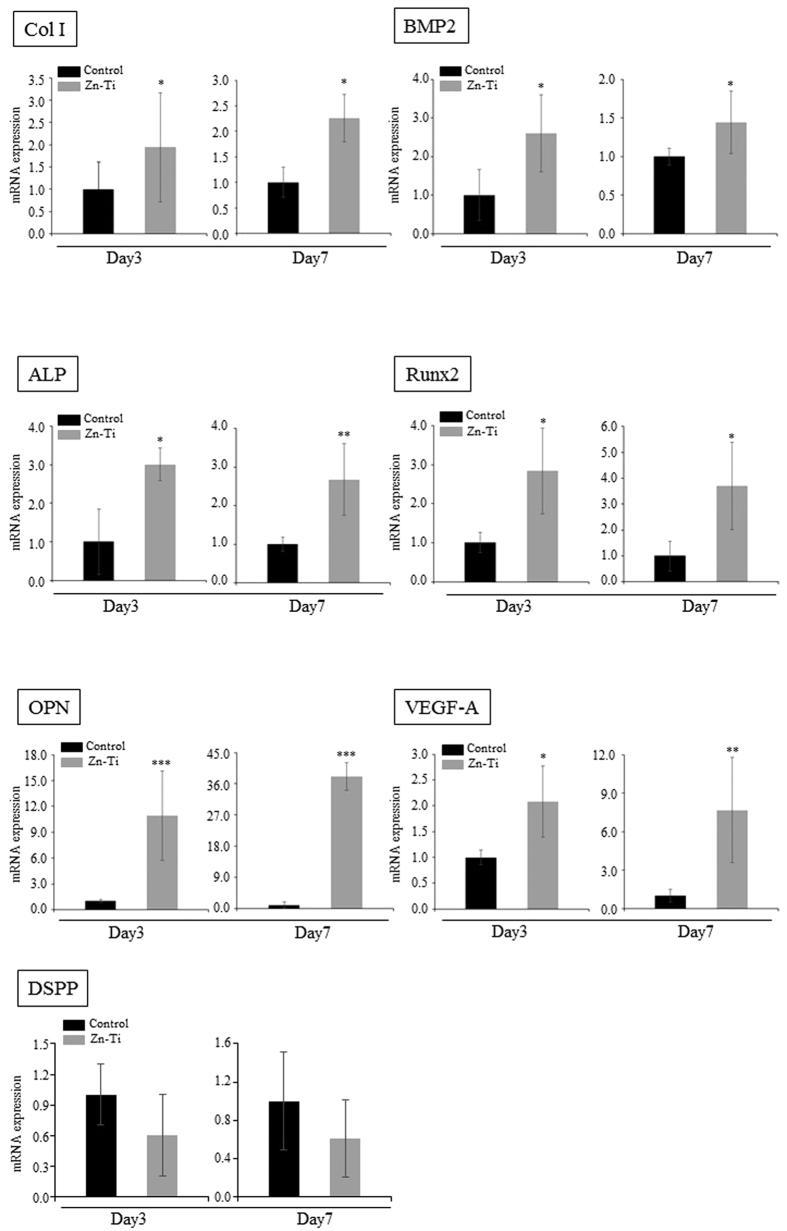
Real-time PCR analysis of Col I, BMP2, ALP, Runx2, OPN, VEGF-A and DSPP in DPSCs on Zn-Ti and control plates. Gene expression is normalized against housekeeping gene GAPDH expression. DPSCs were cultured for 3 and 7 days on Zn-Ti and control plates. Data represent means ± SD (n = 4). *p < 0.05, **p < 0.01, ***p < 0.001, Zn-Ti versus control.

In order to further determine the effects of Zn-Ti on osteoblast differentiation, total RNA was isolated and real-time PCR was performed to measure the gene expression of osteoblast related genes. On days 3 and 7, gene expression levels of col I, BMP2, ALP, Runx2, and OPN were significantly elevated in Zn-Ti when compared with controls. Angiogenesis and blood vessel invasion are the final events in osteoblast differentiation and bone formation. VEGF-A is an angiogenetic factor and VEGF-A mRNA expression is up-regulated in parallel with osteoblast marker gene expression. We also observed that expression levels of VEGF-A were significantly elevated in Zn-Ti groups when compared to those in controls (Fig. 5). Despite the increasing expression levels of osteoblast related genes, Zn-Ti had no effect on DSPP mRNA expression.

### ALP activity

**Figure 6:**
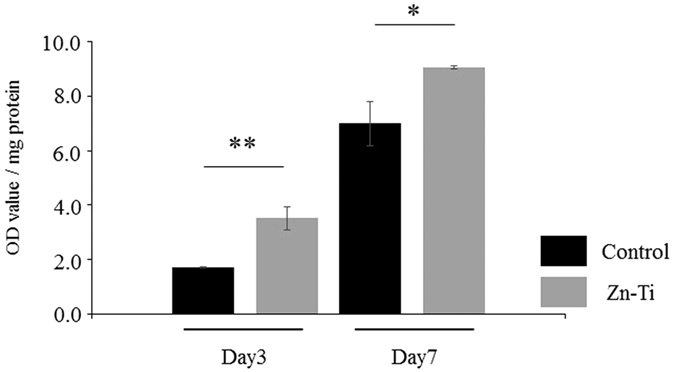
ALP activity of DPSCs cultured on Zn-Ti and control plates. Data represent means ± SD (n = 4). *p < 0.05, **p < 0.01, Zn-Ti versus control.

ALP activity has been used as a marker of early osteoblast differentiation. ALP activity leads to early manifestation of calcification during bone development. In our study, ALP activity of DPSCs on control and Zn-Ti plates was measured on days 3 and 7. There were significant differences in ALP activity between control and Zn-Ti plates, which suggested that Zn-Ti stimulated the early stages of osteoblast differentiation (Fig. 6).

### Protein extraction and Western blot analysis

**Figure 7:**
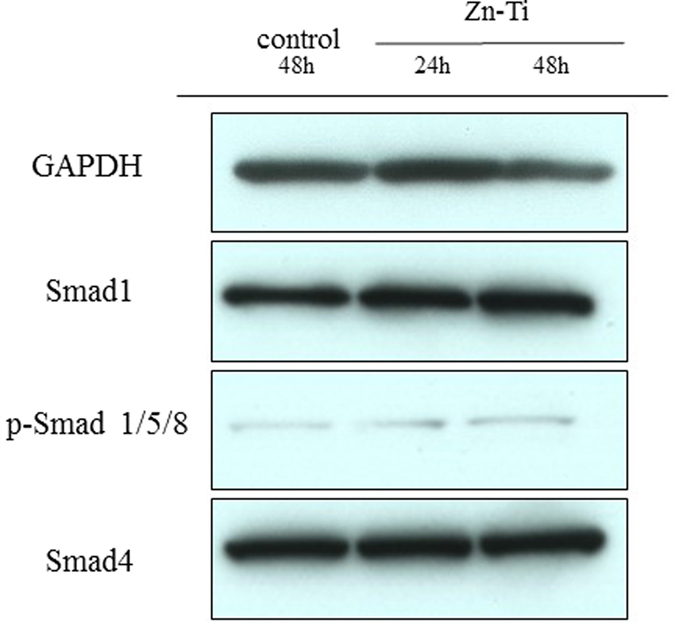
Expression of Smad1, Smad4 and p-Smad1/5/8. Expression of Smad1, p-Smad1/5/8 and Smad4 in DPSCs was analyzed by Western blotting. Protein expression is normalized against GAPDH expression.

There were no obvious differences in Smad 1 and Smad 4 protein expression levels between control and Zn-Ti (Fig. 7). In addition, we found that Zn-Ti induced higher expression of phosphorylated Smad 1/5/8.

### Extracellular matrix (ECM) mineralization

**Figure 8:**
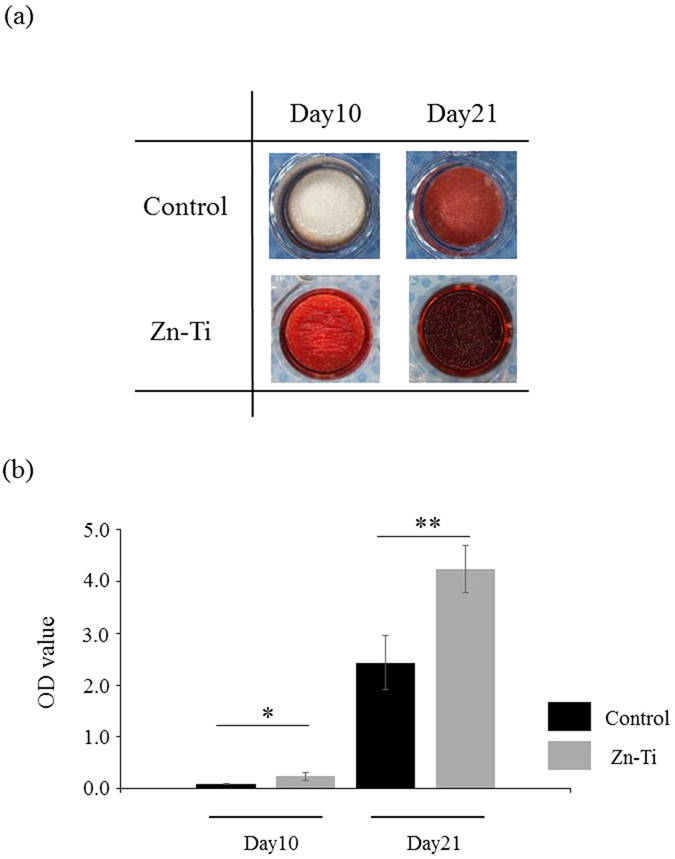
Extracellular matrix mineralization of DPSCs cultured on Zn-Ti and control plates. Extracellular matrix mineralization was assessed by Alizarin Red S (ARS) staining (**a**). After ARS staining, dye was extracted and quantified (**b**). Data represent means ± SD (n = 4). *p < 0.05, **p < 0.01, Zn-Ti versus control.

ECM mineralization was identified by ARS staining of specific calcium binding sites. A representative image of calcified matrix deposition is shown in Fig. 8. ARS staining was followed by elution and quantitation at an absorbance of 405 nm. Zn-Ti showed significantly greater mineralization when compared to controls.

## Discussion

In this study, we explored the use of Zn-Ti as a scaffold for bone regeneration therapies to provide stimulating effects on osteoblast differentiation extracellular matrix mineralization of DPSCs. Autologous bone grafts have been widely used for the treatment of bone defects and nonunion following bone injury. However, some reports have noted morbidity associated with bone harvests for bone grafts, including lasting pain at donor sites^40^,^41^. DPSCs possess higher proliferation capacities than BMSCs and have been identified as a novel population of stem cells that have the capacity of self-renewal and multilineage differentiation. In this study, DPSCs possess mesenchymal stem cell-like properties, including the capacity to regenerate bone, expressing mesenchymal surface molecular markers (CD44-98.6% and CD73-96.7%) and exhibit weak expression of hematopoietic surface markers (CD45-0.5% and CD14-0.5%) (Fig. 3). And the main advantage of using DPSCs from naturally exfoliated deciduous and/or impacted adult wisdom teeth is that can be obtained noninvasively that are routinely extracted in childhood and generally discarded as medical waste without any ethics concerns. In a previous study, we developed Zn-Ti implants and found that the implants exhibited excellent osseointegration in *in vivo* experiments using rabbit femurs^22^. We also found that zinc ions released from the implants stimulated osteoblast marker gene expression and calcium deposition in human bone marrow-derived mesenchymal cells^42^. In this study, Zn-Ti was fabricated by soaking in a solution containing a [Zn(OH)_4_]^2−^ complex. This method incorporates zinc ions into the Ti surface. The present study shows that a porous structure is formed after treatment. Contact angle of the liquid on the substrate surface shows the wettability of such surfaces, and some reports have mentioned that surface roughness and wettability properties are related to physical and chemical properties of the material surface. These factors affect the adhesion, proliferation, and differentiation of osteoblasts, and more hydrophilic surfaces can enhance the osteoblast viability and differentiation^43^,^44^.

Zinc stimulates the differentiation of osteoblasts. However, *in vitro* and *in vivo* studies revealed that an overdose of zinc induced high plasma copper, neurotoxicity, and other poisoning symptoms^45^,^46^. Excess doses of zinc ions may result in various cell fates, such as necrosis, apoptosis, cessation of active growth, and decreased cell viability and proliferation. We detected the amount of released zinc ions from the surface of Zn-Ti plates by incubating plates in 2 ml of PBS. It was estimated that the concentrations of zinc ion releasing from Zn-Ti surface were below the cytotoxic limit that inhibits osteoblast proliferation and differentiation^44^. We also performed cell proliferation assay to evaluate the effects of zinc ion release from Zn-Ti plates on DPSC proliferation and cytotoxicity. As shown in Fig. 3, there were no cytotoxic effects of zinc ion release from the Zn-Ti surface.

Osteoblast-related gene expression is one of the major bone formation events induced by immature and mature osteoblasts. In this study, gene expression levels of Col I, BMP2, ALP, Runx2, and OPN were up-regulated on Zn-Ti. Col I expression is one of the most widely recognized early markers for osteoblast differentiation^47^. BMP2 is widely known to induce osteoblast differentiation^48^ and Runx2 is an osteogenic transcription factor that induces the process of BMSC differentiation towards osteoblasts^49^. OPN is a mature osteoblast marker gene and is expressed in the late mineralization and remodeling phase. Some reports have mentioned that Runx2 induces the expression of OPN, and expression patterns of Runx2 and OPN were similar during osteoblast differentiation and bone formation^50^,^51^,^52^. ALP gene expression and ALP activity induce early manifestation of calcification during osteoblast development. DPSCs cultured on Zn-Ti also exhibit higher gene expression and ALP activity than those of controls, which suggests that the presence of zinc ion releasing from Zn-Ti stimulated the early stages of osteoblast differentiation. VEGF-A is an angiogenic factor and VEGF-A mRNA expression is up-regulated during terminal differentiation in parallel with osteoblast marker gene expression^53^,^54^, and we found gene expression levels of VEGF-A were up-regulated on Zn-Ti.

TGF-beta/BMP signals induce proliferation of various cell types and differentiation such as angiogenesis and osteogenesis. Particularly in osteoblast differentiation, TGF-beta/BMP signaling stimulates osteoprogenitor proliferation and differentiation through the Smad 1/5/8 pathway^55^,^56^,^57^. Our results also suggested that DPSC culture on Zn-Ti induces protein levels of phosphorylated Smad1/5/8 (Fig. 7). These results strongly support the hypothesis that zinc ions released from Zn-Ti play a key role in DPSC osteoblast differentiation.

ECM mineralization is the eventual phenotypical expression of osteoblast differentiation and depends on cell culture conditions. In this study, we performed ARS staining, and Zn-Ti markedly increased ECM mineralization at 10 and 21 days in DPSCs when compared with controls.

Based on these results, a combination of Zn-Ti and DPSCs would promote osteoblast differentiation *in vitro*. Our results also indicate the future possibility of clinical application of Zn-Ti and DPSCs in various bone diseases and bone defects. Hence, further detailed *in vitro* and *in vivo* studies are necessary.

## Methods

### Fabrication of zinc-modified titanium (Zn-Ti) plates

Titanium plates 10 × 10 × 1 mm were fabricated from commercially pure grade 2 titanium (purity > 99.85%). Ti plates were mirror polished and then ultrasonically cleaned with ethanol and deionized water, followed by drying at 70 °C for 3 hours in air to remove contaminants related to manufacturing and handling. An alkali solution containing the [Zn(OH)_4_]^2−^ complex was prepared by dissolving 14.85 g of zinc nitrate hexahydrate (Nacalai Tesque, Japan) and 24.00 g of sodium hydroxide (Nacalai Tsque) in deionized water to give a 100 ml solution with a zinc ion concentration of 0.5 M. Ti plates were soaked in the solution at 60 °C for 24 h. At the end of the soaking period, plates were washed thoroughly for 5 min with running deionized water and were subjected to dry-heat sterilization carried out at 180 °C for 30 min. Untreated plates were used as controls.

### Surface characterization

Sample surfaces were observed and by field emission scanning electron microscopy (FESEM) (S-4500; Hitachi, Japan) and confocal laser scanning microscopy (VK 9700; Keyence, Japan). Surface roughness was also measured by a confocal laser scanning microscope. The surface wettability of controls and Zn-Ti was measured by contact angle of 3 μL H_2_O. Six samples were used for each measurement.

### Zinc ion release

The Zn-Ti samples were soaked in 2 ml of phosphate buffered saline (PBS) in a sealed bottle at 37 °C for 1 day, removed, and then immersed again in 2 ml of PBS. This process was repeated. The released zinc ion concentrations in PBS at day 1, 3, 7 and 14 were measured by inductively coupled plasma atomic emission spectrometry (ICP-AES).

### Cell culture

Dental pulp tissue from human deciduous teeth was kindly provided by Toshihiro Sugiyama (Department of Biochemistry, Akita University Graduate School of Medicine) and written informed consent was obtained from the participant. All experimental protocols were approved by the Ethics Committee of Yamagata University School of Medicine, and carried out in accordance with the approved guidelines. Human DPSCs were isolated, as reported previously^58^. DPSCs were cultured in standard medium consisting of Dulbecco’s Modified Eagle’s Medium (DMEM) (D6046; Sigma, USA) supplemented with 10% fetal bovine serum (Gibco, USA) and 1% penicillin/streptomycin (P0781; Sigma) at 37 °C in a humidified atmosphere of 5% CO_2_ in air. To characterize DPSCs, immunophenotyping profiling was performed at passage 3 in order to detect specific cell surface antigen sets for mesenchymal stem cells using flow cytometry. Cells were washed with PBS and detached with trypsin/EDTA (T4049; Sigma). The following antibodies were used: FITC-conjugated mouse CD44, CD73, CD45, and CD14 (Biolegend, USA), with an isotype-matched negative control IgG1 (Biolegend) (CD44, CD73, CD45) or IgG2a (Biolegend) (CD14). Quantities of 2.6 × 10^6^ cells were incubated with primary antibody for 30 min on ice. Fluorescence intensity was measured on a flow cytometer (FACS Canto II; BD, USA) and data were analyzed using FACS Diva version 6. 1. 3 (BD).

For osteoblast differentiation, DPSCs were provided with osteogenic medium consisting of standard medium containing 10 mM β-glycerophosphate (Sigma), 0.28 mM ascorbic acid 2-phosphate (Wako Pure Chemical; Japan), and 100 nM dexamethasone (Sigma). Cells were seeded on the Ti surface (1500 cells/cm^2^) for further experiments, and medium was changed every 2 days.

### Cell proliferation assay

Cells were seeded at a density of 1500 cells/cm^2^ on control and Zn-Ti samples and were cultured at 37 °C in a humidified atmosphere with 5% CO_2_.

Proliferation of seeded cells cultured for 1, 3, 5, and 7 days was detected using the CellTiter 96 AQ One Solution Cell Proliferation Assay Kit (Promega, USA) in accordance with the manufacturer’s instructions. The absorbance of each sample was observed at 562 nm using a plate reader (Varioskan Frash 2.4; Thermo science, USA).

### Expression of osteoblast- and odontoblast-related genes

Expression levels of osteoblast-related genes after 3 and 7 days of culture on control and Zn-Ti plates were measured using real-time PCR. Total RNA was isolated using RNA iso (Takara Bio, Japan) according to the manufacturer’s instructions. RNA concentrations were calculated by absorption ratio (OD_260_/OD_280_) using an e-spect (BM Equipment, Japan). First-strand cDNA was synthesized using a PrimeScript RT reagent Kit (Perfect Real Time) (Takara Bio) according to the manufacturer’s instructions. Real-time PCR was performed with a CFX96 (Bio Rad, USA) using SYBR Premix Ex Taq II (Takara Bio). Osteoblast-related genes, including type I collagen (Col I), bone morphogenetic protein 2 (BMP2), alkaline phosphatase (ALP), runt-related transcription factor 2 (Runx2), osteopontin (OPN), and vascular endothelial growth factor A (VEGF-A) were amplified using custom-made forward and reverse primers. And we also investigated the dentin sialophosphoprotein (DSPP). The following cycling parameters were used: 95 °C for 30 s; 40 cycles of 95 °C for 5 s and 60 °C for 30 s; followed by a dissociation stage. Relative expression ratio of the markers was calculated based on the (ddct) comparative threshold cycle (CT) method. Calculated values ware normalized against the internal control glyceraldehyde-3-phosphate dehydrogenase (GAPDH).

### Alkaline phosphatase activity

At days 3 and 7, alkaline phosphatase (ALP) activity of the cells grown on control and Zn-Ti plates was determined using p-nitrophenylphosphate Disodium. Cells were washed twice with PBS and lysed in Triton X (Sigma). ALP activity was assayed using p-nitrophenylphosphate Disodium (LabAssay ALP; Wako, Japan), in accordance with the manufacturer’s instructions. After 30 min of incubation, 50 mM NaOH was added to stop the reaction. The presence of p-nitrophenylphosphate was measured at 410 nm. Total cellular protein was determined using BCA assay (Takara BCA protein assay Kit; Takara Bio). Enzyme activity was normalized against total cellular protein.

### Protein extraction and Western blot analysis

After 24 h (Zn-Ti) and 48 h (control and Zn-Ti) of culture, cell pellets were lysed in ice-cold RIPA buffer (25 mM Tris-HCl (pH 7.5), 150 mM NaCl, 1% (w/v) Nonidet P-40, 0.5% Sodium Deoxycholate, 0.1% SDS, and 1/100 (v/v) Protease Inhibitor Cocktail (Sigma) and Phosphatase Inhibitor Cocktail (Nacalai Tesque) and were incubated at 4 °C for 30 min with vortexing. Lysates were then passed 5 times through a 27 G needle. After centrifugation at 18,000× g, 4 °C, for 15 min, supernatant was collected as protein extracts and protein concentration was measured using a BCA Protein Assay Kit (Thermo Scientific) with BSA as a standard. Protein extracts were mixed with SDS sample buffer containing dithiothreitol and were heated at 45 °C for 30 min, followed immediately by cooling on ice. An aliquot of 15 μg of protein from each sample was separated by SDS-PAGE on 5–20% gels (ATTO, Japan), and was transferred to a PVDF membrane (BioRad). After blocking in 0.05% Triton-TBS containing 1% BSA (Nacalai Tesque) at room temperature for 60 min, the membrane was incubated at room temperature overnight with primary antibody (Smad1, Phospho-Smad1/5/8, Smad4; Cell Signaling Technology (CST)). Subsequently, excess antibody was washed away with 0.05% TBST and the membrane was incubated with HRP-linked anti-rabbit IgG antibody (CST) at room temperature for 2 hours. After again washing away excess antibody, membranes were incubated with SuperSignal West Pico Chemiluminescent Substrate (Thermo Scientific) at room temperature for 5 min, and signals were detected using X-ray film. After exposure, membranes were incubated in stripping buffer (62.5 mM Tris-HCl (pH 7), 1% SDS, 100 mM 2-mercaptoethanol) at 37 °C for 30 min to strip off remaining antibody. Membranes were then incubated with Anti-GAPDH (AbD Serotec) and HRP-linked anti-mouse IgG antibody (CST) to detect GAPDH protein as a loading control.

### Extracellular matrix (ECM) mineralization

At days 10 and 21, cells grown in osteogenic medium on control and Zn-Ti plates were fixed in 10% paraformaldehyde (PFA) for 30 min at room temperature and were treated with Alizarin Red S (ARS) (Iwai Chemicals, Japan) for 30 min. Calcium content was detected using a colorimetric method, 2% formic acid (Iwai Chemicals) were added to each well and the plate was incubated at room temperature for 10 min under shaking. This solution was transferred to a new tube and ARS concentration was determined by measuring the absorbance at 405 nm.

### Statistical analysis

Data are expressed as means ± SD. All experiments were repeated at least three times, and Student’s t-test was performed to test the significance of results. Data were considered statistically significant when P < 0.05.

## Additional Information

**How to cite this article**: Yusa, K. *et al*. Zinc-modified titanium surface enhances osteoblast differentiation of dental pulp stem cells *in vitro. Sci. Rep.* **6**, 29462; doi: 10.1038/srep29462 (2016).

## Figures and Tables

**Table 1:** Contact angle and surface roughness of Zn-Ti and control.

	Control	Zn-Ti
Contact angle	89.25 ± 0.69°	10.00 ± 0.45°
Surface roughness	0.20 ± 0.04 μm	0.33 ± 0.05 μm
